# Development and assessment of a telesonography system for musculoskeletal imaging

**DOI:** 10.1186/s41747-021-00227-z

**Published:** 2021-07-27

**Authors:** Mohammed Obaid, Qianwei Zhang, Scott J. Adams, Reza Fotouhi, Haron Obaid

**Affiliations:** 1 Department of Physics and Astronomy, Faculty of Science, University of British Columbia, 6224 Agricultural Road, Vancouver, BC V6T 1Z1 Canada; 2 Department of Mechanical Engineering, College of Engineering, University of Saskatchewan, 57 Campus Drive, Saskatoon, SK S7N 5A9 Canada; 3 Department of Medical Imaging, College of Medicine, University of Saskatchewan, Royal University Hospital, 103 Hospital Drive, Saskatoon, SK S7N 0W8 Canada

**Keywords:** Musculoskeletal system, Robotics, Telemedicine, Teleradiology, Ultrasonography

## Abstract

**Background:**

Telesonography systems have been developed to overcome barriers to accessing diagnostic ultrasound for patients in rural and remote communities. However, most previous telesonography systems have been designed for performing only abdominal and obstetrical exams. In this paper, we describe the development and assessment of a musculoskeletal (MSK) telesonography system.

**Methods:**

We developed a 4-degrees-of-freedom (DOF) robot to manipulate an ultrasound probe. The robot was remotely controlled by a radiologist operating a joystick at the master site. The telesonography system was used to scan participants’ forearms, and all participants were conventionally scanned for comparison. Participants and radiologists were surveyed regarding their experience. Images from both scanning methods were independently assessed by an MSK radiologist.

**Results:**

All ten ultrasound exams were successfully performed using our developed MSK telesonography system, with no significant delay in movement. The duration (mean ± standard deviation) of telerobotic and conventional exams was 4.6 ± 0.9 and 1.4 ± 0.5 min, respectively (*p* = 0.039). An MSK radiologist rated quality of real-time ultrasound images transmitted over an internet connection as “very good” for all telesonography exams, and participants rated communication with the radiologist as “very good” or “good” for all exams. Visualisation of anatomic structures was similar between telerobotic and conventional methods, with no statistically significant differences.

**Conclusions:**

The MSK telesonography system developed in this study is feasible for performing soft tissue ultrasound exams. The advancement of this system may allow MSK ultrasound exams to be performed over long distances, increasing access to ultrasound for patients in rural and remote communities.

## Key points

- Soft tissue musculoskeletal ultrasound exams were successfully performed remotely using the telesonography system.
- Diagnostic ability of the musculoskeletal telesonography system was similar to conventional ultrasound.
- Musculoskeletal telesonography may increase access to subspecialised ultrasound in remote communities.

## Background

Ultrasound imaging is increasingly used to assess musculoskeletal (MSK) pathology, with broad applications ranging from assessment of soft-tissue masses to assessment of tendon pathology, for example [1]. Ultrasound imaging is a cost-effective imaging modality for initial assessment of soft-tissue masses and is widely available in most urban centres [2]. However, access to MSK ultrasound imaging is limited for many patients in rural and remote communities due to a lack of MSK sonographers and radiologists in these communities. As a result of limited access to ultrasound services, patients in many rural and remote communities must travel to a larger centre for imaging, potentially leading to delays in diagnosis, patient inconvenience, and added costs for patients to travel [3, 4].

The remote provision of specialised MSK ultrasound services using telerobotic technology holds the potential to increase access to ultrasound imaging for patients in rural and remote communities. Telesonography systems (telerobotic ultrasound systems) allow sonographers or radiologists based at a central site, such as an urban ultrasound clinic or tertiary hospital, to remotely perform ultrasound while patients stay in their home communities [5–8]. Two dominant designs have emerged in the literature for telesonography systems: spherical wrist-like designs and jointed-arms [9]. One of the dominant, now commercialised, telesonography systems which uses a spherical wrist-like design is the MELODY system (Société AdEchoTech, Naveil, France) [10]. This system is a 3-degrees-of-freedom (DOF) robot that contacts the patient’s body through a ring-like frame. While well suited to scanning anatomic regions of a relatively large surface area to perform abdominal and obstetrical exams [7, 11], for example, it is not as well suited to scanning an extremity. Medirob Tele (Medirob AB, Skellefteå, Sweden [12]) and ROSE (Sensing Future Technologies, Coimbra, Portugal [13]) are both commercialised 6-DOF robots for telesonography which have a jointed-arm design, and these robots may be particularly well suited for scanning joints such as the shoulder. Another strategy that has been used to facilitate remote ultrasound imaging is adding small motors to ultrasound probes to allow users to remotely control their movement. Arbeille et al. [14] equipped a convex array ultrasound probe with two motors to tilt and rotate the probe, respectively, and equipped a linear array probe with a motor to tilt the probe. While motorised linear array probes were used to image muscles in a limited number of patients, a disadvantage is that exams heavily relied on an assistant at the patient site who assisted with all other movements of the ultrasound probe (*e.g.,* translation, rotation, and rocking) to complete the exam [14].

In this paper, we describe the development of a 4-DOF telesonography system to allow experts (radiologists and sonographers) to remotely control movements of an ultrasound probe and remotely perform MSK ultrasound exams without an assistant at the patient site. This study assesses the technical feasibility, safety, diagnostic quality, and patient and radiologist satisfaction of using this MSK telesonography system to remotely perform MSK ultrasound exams. The telerobotic ultrasound system described in this paper may pave the way for patients living in underserved rural and remote communities to access subspecialty MSK ultrasound imaging expertise in their home community.

## Methods

### Development of the MSK telesonography system

**Fig. 1:**
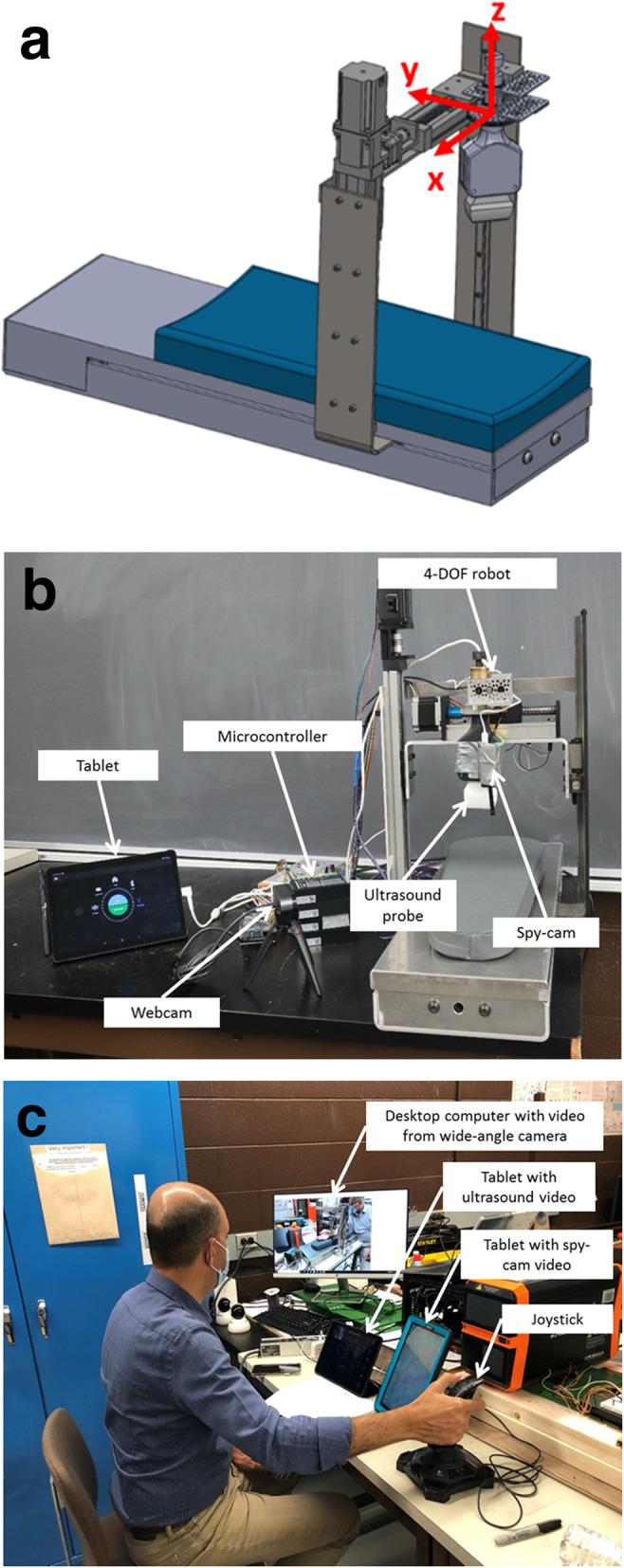
Schematic (**a**) and prototype (**b**) of the 4-degrees-of-freedom musculoskeletal telesonography system at the patient site. Setup of the system at the master site (**c**), with the radiologist controlling movements of the ultrasound probe via a joystick

An MSK telesonography system was developed to allow radiologists to remotely scan patients’ upper and lower limbs using a master-slave robotic system. The system developed is a 4-DOF robot manipulator. The robot includes three linear and one revolute joints (Fig. 1a). Dimensions of this device are approximately 30” × 10” × 24”. The ultrasound probe can move along *x*, *y*, and *z* axes and rotate about the *z*-axis (Fig. 1a). The motion in each direction is precisely controlled through stepper motors and corresponding controllers; controllers set limits for maximum displacement of the ultrasound probe, protecting patients from injury. The accuracy of linear motion is approximately 0.01 in. (0.25 mm), and the accuracy of revolute motion is 0.01°. The main frame of the device is made of aluminium to reduce weight and to protect the system from corrosion. An arm pad on the top surface allows patients to rest their arm securely during scanning.

This device has the following features:

1. The probe can move over a distance of 200 mm along the *y*-axis of the upper or lower extremity;
2. The probe can move over a distance of 150 mm along the *x*-axis of the upper or lower extremity;
3. The probe rotates between −180° to 180° about the *z*-axis (perpendicular to the extremity); and
4. The sensor head touches the patient’s skin with sufficient but safe load for a clear image.

The patient side (slave station, Fig. 1b) and radiologist side (master station, Fig. 1c) are equipped for controlling the probe motion and streaming live video. On the patient side, an ultrasound probe (Lumify, Philips, Amsterdam, The Netherlands [15]) is connected to a tablet, and an Android application (Reacts, Innovative Imaging Technologies, Montreal, Canada [16]) transmits real-time ultrasound images to the radiologist side or other users [16]. To move the ultrasound probe, the radiologist manipulates a joystick along 4 DOF. A PC, connected to the joystick by universal serial bus, runs a programme to read and send motion commands from the radiologist side to a Raspberry Pi computer (Raspberry Pi Foundation, Cambridge, UK [17]) which controls the robot at the patient side. The radiologist is able to control the axis of movement of the ultrasound probe using several buttons on the joystick. Displacement of the joystick forward or backward corresponds to linear translation of the probe along the *y*-axis; the same action performed while holding a central button on the joystick corresponds to translation along the *z*-axis (up and down). Without manipulating the joystick itself, two buttons also located on the joystick are used to translate the probe along the *x*-axis (left and right), and two buttons below the joystick control the angle of rotation of the probe about the *z*-axis. The radiologist manually adjusts the load applied by the robot (pressure of the ultrasound transducer on the patient) by moving the ultrasound probe along the *z*-axis. As pressure of the ultrasound probe on the patient is manually controlled by the radiologist, there are no pre-set load selections for scanning different anatomic regions. The motor controller limits the maximum displacement, ensuring patient safety. The radiologist can view the patient and the position of the ultrasound probe via video from three cameras located at the patient side. These cameras include (1) a camera which is built into the tablet, displaying a medium angle view of the patient and robot; (2) a webcam displaying a wide angle image of the patient and robot; and (3) a small spy camera mounted on the probe holder of the robot, displaying a top-down view of the anatomic area to be examined (Fig. 1b). The patient can communicate with the radiologist through the tablet video link.

### Clinical assessment

This clinical study was approved by the University of Saskatchewan Research Ethics Board, and written informed consent was obtained from each participant. Ultrasound exams were performed using two methods: conventional, whereby a radiologist and patient were located in the same room and the ultrasound probe was manually operated by the radiologist; and robotic, whereby the radiologist remotely operated the MSK telesonography system using the joystick in a room separate from the patient. Robotic and conventional exams were performed by the same radiologist using the same ultrasound probe. Robotic exams were performed after conventional exams in all cases. All participants were presumed to be healthy with no known MSK pathology.

After each exam, each participant was invited to complete a survey comprised of Likert-scale items to assess patient experience in two domains: (1) comfort/discomfort during the exam and (2) ability to communicate with the radiologist during the exam. A 5-point Likert scale was used with descriptors including “very comfortable,” “comfortable,” “neutral,” “uncomfortable,” and “very uncomfortable” for a question regarding participant comfort/discomfort, and “very good,” “good,” “neutral,” “poor,” and “very poor” for a question regarding communication. The survey also included open-ended questions related to the participant’s experience more generally, providing an opportunity for free-text responses from participants. Similarly, the radiologist assessed each exam in four domains: (1) image quality, (2) ergonomics, (3) convenience, and (4) ability to communicate with the patient. A 5-point Likert scale with descriptors “very good,” “good,” “neutral,” “poor,” and “very poor” was used to assess each domain.

An MSK radiologist subsequently assessed the quality of each image on the basis of visualisation of anatomic structures in the forearm. Radiologists were blinded to all participants’ identifiers and were blinded to whether images were obtained telerobotically or conventionally. Using a standardised reporting form, the radiologist assessed whether muscles, myotendinous junctions, tendons, neurovascular bundles, and bones were completely (adequately) documented, partially documented, or not documented based on the representative images.

### Statistical and thematic analysis

Statistical analysis was performed using IBM SPSS Statistics 26.0 (IBM, Armonk, New York) and Stata 15.1 (StataCorp, College Station, Texas). Duration of telerobotic and conventional exams was summarised using means and standard deviations and compared using the Wilcoxon signed-rank test. Frequencies and proportions of participant and radiologist responses to Likert-scale items were determined and compared using Fisher’s exact test. Visualisation scores for each anatomic structure were also summarised by frequencies and proportions, and visualisation scores for telerobotic and conventional methods were compared using Fisher’s exact test. All *p*-values less than 0.05 were considered statistically significant.

Free-text responses from participants were analysed using thematic analysis, an established methodology from the social sciences to identify common themes among participant responses [18]. Responses were analysed with a view towards identifying potential areas for future development of the MSK telesonography system.

## Results

Five participants (ten forearms) underwent telerobotic and conventional ultrasound, and transverse images of the proximal, mid, and distal aspects of each forearm were captured resulting in twelve images per participant. All exams were performed successfully, with no significant delay in probe movement reported by the radiologist. No safety concerns were reported during telerobotic or conventional exams. The duration (mean ± standard deviation) of telerobotic and conventional exams (for bilateral forearms) was 4.6 ± 0.9 and 1.4 (±0.5) min, respectively (*p* = 0.039).

Radiologist assessment scores were “very good” for all (100%) exams performed conventionally across all four domains (patient communication, image quality, ergonomics, and convenience). For telesonography exams, all (100%) exams were scored as “very good” in the domains of patient communication and image quality, while exams for three (60%) participants were scored as “very good” and exams for two (40%) participants were scored as “good” in the domains of ergonomics and convenience (Table 1).

**Table 1:** Radiologist assessment of robotic and conventional exams

	Robotic, n (%)	Conventional, n (%)	p*
**Patient communication**			–
Very good	5 (100)	5 (100)	
Good	0 (0)	0 (0)	
Neutral	0 (0)	0 (0)	
Poor	0 (0)	0 (0)	
Very poor	0 (0)	0 (0)	
**Image quality**			–
Very good	5 (100)	5 (100)	
Good	0 (0)	0 (0)	
Neutral	0 (0)	0 (0)	
Poor	0 (0)	0 (0)	
Very poor	0 (0)	0 (0)	
**Ergonomics**			0.444
Very good	3 (60)	5 (100)	
Good	2 (40)	0 (0)	
Neutral	0 (0)	0 (0)	
Poor	0 (0)	0 (0)	
Very poor	0 (0)	0 (0)	
**Convenience**			0.444
Very good	3 (60)	5 (100)	
Good	2 (40)	0 (0)	
Neutral	0 (0)	0 (0)	
Poor	0 (0)	0 (0)	
Very poor	0 (0)	0 (0)	

*Based on Fisher’s exact test. As exams of the right and left forearms were performed immediately after each other, data represent combined assessments of both exams (right and left) for each participant

All participants indicated that conventional ultrasound exams were “very comfortable” or “comfortable.” In comparison, four (80%) participants indicated telesonography exams were “very comfortable” or “comfortable,” with one (20%) participant indicating that the telesonography exam was “neither comfortable nor uncomfortable.” No differences were observed in participants’ assessment of the ability to communicate with the radiologist for conventional and telerobotic exams (Table 2).

**Table 2:** Participant assessment of robotic and conventional ultrasound exams

	Robotic, n (%)	Conventional, n (%)	p*
**Comfort/discomfort during the ultrasound exam**			0.524
Very comfortable	1 (20)	3 (60)	
Comfortable	3 (60)	2 (40)	
Neither comfortable nor uncomfortable	1 (20)	0 (0)	
Uncomfortable	0 (0)	0 (0)	
Very uncomfortable	0 (0)	0 (0)	
**Ability to communicate with the radiologist during the ultrasound exam**			–
Very good	4 (80)	4 (80)	
Good	1 (20)	1 (20)	
Neutral	0 (0)	0 (0)	
Poor	0 (0)	0 (0)	
Very poor	0 (0)	0 (0)	

*Based on Fisher’s exact test

**Fig. 2:**
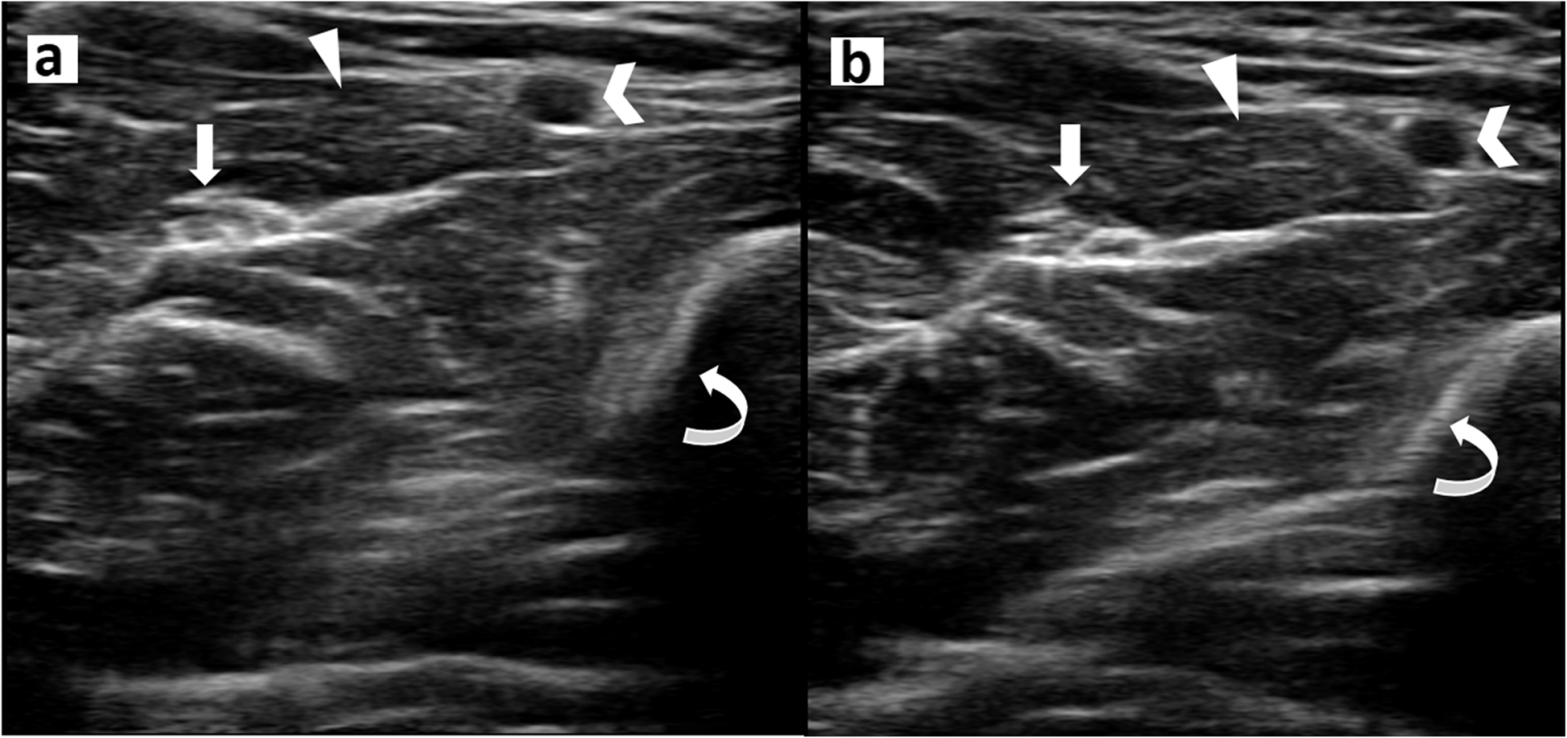
Transverse ultrasound images of the left mid forearm obtained through conventional (**a**) and robotic (**b**) techniques which demonstrate median nerve (arrow), flexor digitorum superficialis muscle (arrow head), radial artery (chevron), and radius (curved arrow)

Visualisation of anatomic structures was similar between robotic and conventional images based on the radiologist’s blinded assessment, as presented in Table 3. No statistically significant differences were observed in the proportion of muscles, myotendinous junctions, tendons, neurovascular bundles, and bones which were completely (adequately) documented, partially documented, or not documented based on the representative images (Fig. 2).

**Table 3:** Visualisation of anatomic structures on robotic *versus*. conventional images

	Robotic, n (%)	Conventional, n (%)	p*
**Proximal forearm**			
Muscle			–
Completely documented	9 (90)	9 (90)	
Partially documented	1 (10)	1 (10)	
Not documented	0 (0)	0 (0)	
Neurovascular bundle			0.325
Completely documented	3 (30)	4 (40)	
Partially documented	7 (70)	4 (40)	
Not documented	0 (0)	2 (20)	
Bone			0.243
Completely documented	3 (30)	7 (70)	
Partially documented	1 (10)	1 (10)	
Not documented	6 (60)	2 (20)	
**Mid forearm**			
Myotendinous junction			–
Completely documented	10 (100)	10 (100)	
Partially documented	0 (0)	0 (0)	
Not documented	0 (0)	0 (0)	
Neurovascular bundle			1.000
Completely documented	8 (80)	9 (90)	
Partially documented	1 (10)	1 (10)	
Not documented	1 (10)	0 (0)	
Bone			0.386
Completely documented	2 (20)	4 (40)	
Partially documented	4 (40)	1 (10)	
Not documented	4 (40)	5 (50)	
**Distal forearm**			
Tendon			0.582
Completely documented	7 (70)	9 (90)	
Partially documented	3 (30)	1 (10)	
Not documented	0 (0)	0 (0)	
Neurovascular bundle			0.582
Completely documented	8 (80)	7 (70)	
Partially documented	1 (10)	3 (30)	
Not documented	1 (10)	0 (0)	
Bone			0.721
Completely documented	9 (90)	7 (70)	
Partially documented	0 (10)	2 (20)	
Not documented	1 (10)	1 (10)	

*Based on Fisher’s exact test

Thematic analysis of participants’ free-text responses to survey questions regarding their experience during telerobotic ultrasound exams identified themes related to ergonomics, communication with the radiologist, and movement of the telerobotic ultrasound system as potential areas for future development. Related to ergonomics, participants felt that the table could be positioned higher to minimise discomfort at the shoulder and elbow during scanning. Participants felt that the forearm could be nicely positioned in the frame of the telerobotic ultrasound system, though the pad on which to rest the forearm could be made more comfortable. Related to communication, one participant noted that a larger monitor would allow patients to better see and communicate with the radiologist during the ultrasound exam. Finally, pertaining to movement of the telesonography system, participants commented that pressure on the forearm was inconsistent and movement of the telesonography system could be smoother. One participant specifically commented that the duration of the telesonography exam was much longer than the conventional ultrasound exam.

## Discussion

In this study, we described the development of a telesonography system to perform MSK ultrasound exams. As an advancement on previous telesonography systems which were developed for abdominal and obstetrical exams, the unique design of the telesonography system described in this paper is ideally suited to scanning the extremities, without requiring an assistant at the patient site to manoeuvre the device. Assessment of the telesonography system for scanning the forearm demonstrated similar diagnostic ability compared to conventional ultrasound for assessment of anatomic structures of the forearm. Radiologists found that image quality while performing telesonography exams was very good, and radiologists and patients found that communication with each other was sufficient, despite the ultrasound video being transmitted over an internet connection and despite communicating with each other via video conferencing.

The study also identified domains (namely, ergonomics and convenience for the radiologist) in which the conventional technique was more favourable than the robotic technique. These areas can inform further development of MSK telesonography systems. Using a more user-friendly joystick or a fictive probe (particularly one that directly replicates the motions of scanning patients conventionally) and incorporating haptic feedback may improve the convenience of performing telesonography exams from radiologists’ perspectives. Additionally, adding a load sensor would allow radiologists to monitor the load applied by the ultrasound probe. The addition of a degree of freedom to allow tilting of the ultrasound probe, for example, would allow radiologists to efficiently minimise ultrasound artefacts such as anisotropy. Further work should also explore the potential for radiologists to remotely control ultrasound unit settings, such as gain or depth, via the Reacts or equivalent software.

This study builds on previous work in the area of telesonography. Current telesonography systems require an assistant at the patient-site to control gross movements of the frame for the telesonography system [19] or some movements of the ultrasound probe itself [14]. In contrast, an advantage of the MSK telesonography system developed in this study is that no assistant is required to assist in facilitating any movements of the ultrasound probe, providing radiologists with the ability to control the ultrasound probe independently. In comparison to spherical wrist-like designs [10, 20–23] and jointed-arm designs [12, 13, 24, 25] which are currently dominant in the telesonography literature, to our knowledge, the design proposed in this paper is unique and may be ideally suited to scanning the soft tissues of the upper and lower extremities. Although in our study the duration of telesonography exams was significantly longer than for conventional exams, similar differences in exam duration have been found in prior studies comparing telerobotic and conventional ultrasound for abdominal exams [7, 26]. A trend of shorter scan time towards the end of our trial suggests that there was a learning curve in manoeuvring the joystick, and scan times may continue to decrease with additional experience in using the telesonography system.

Telesonography may be an important component of an MSK ultrasound service delivery model for patients in rural and remote communities in the future. As telesonography is considered for future integration into health systems, it will be important to consider the costs involved with telesonography, including hardware costs and increased sonographer costs or radiologist costs (opportunity costs) due to longer exam durations. A study from Sweden which trialled a telesonography system for echocardiography found that costs related to conventional ultrasound and telesonography were similar from the perspective of the health system, but costs of a service delivery model incorporating telesonography were less from the perspective of patients due to reduced travel costs [27]. Further research should include a cost analysis of telesonography for MSK ultrasound imaging. Principles of health equity for patients in rural and remote communities and the need for timely assessment of soft-tissue masses (which the MSK telesonography system designed in this study is best suited for) may help justify any incremental costs of telesonography compared to conventional ultrasound.

A strength of this study is that the same ultrasound probe was used for both conventional and telerobotic scanning, allowing differences in visualisation to be attributed to the method of scanning (conventional *versus* telerobotic) rather than the ultrasound probe. The same radiologist performed both conventional and telesonography exams for each patient, also allowing for consistency between conventional and telerobotic approaches. Additionally, the mixed-methods study design allowed greater depth of analysis and understanding of participant experiences than could be achieved if using a quantitative study design alone, and identified specific areas for further development of MSK telesonography systems.

There are also a few limitations to this study. First, the study assessed the diagnostic ability of the telesonography system to assess only normal anatomy. Future work should also assess the potential for the telesonography system to assess pathologic findings (*e.g.,* soft-tissue masses). Second, only one radiologist scanned all participants. While this brought consistency across robotic and conventional exams, future assessment across multiple radiologists and sonographers would increase generalizability of results. Third, while the radiologist and the participant were in separate rooms during telerobotic ultrasound exams, the proximity between the participant and radiologist (in the same building) did not allow for assessment of lag which may result from an internet connection which extends over long distances typical of communication between remote communities. Further research should trial the MSK telesonography system with patients located in communities which are separated by significant distances from the radiologist site, including trials in communities which have variable bandwidth. An additional limitation is the small sample size which limits the statistical comparisons between telerobotic and conventional methods of scanning. Finally, our study was limited to assessing the feasibility of using the telesonography system for imaging the upper extremity. The parameters for the range of motion of the telerobotic ultrasound system may also allow for scanning of the soft tissues of the lower extremity, and further work should explore this additional application for the MSK telesonography system.

In conclusion, this study demonstrates the technical feasibility, safety, and diagnostic ability of using the MSK telesonography system which we developed to assess the soft tissues of the extremities. Future work will address some of the limitations of the system as identified by participants and radiologists and will assess this technology in patients with known pathology. We envision the development of a network of telesonography systems placed in communities without regular access to subspecialised MSK ultrasound imaging to allow radiology groups to increase the reach of their practice and support efforts to improve access to imaging for patients in remote communities. Advancing this technology may improve patient experience and satisfaction, reduce time to diagnosis, and help achieve better health equity in rural and remote communities.

## Data Availability

Available upon request.

## Abbreviations

DOF: Degrees of freedom
MSK: Musculoskeletal
